# Synergistic LPCVD and PECVD Growth of β-Ga_2_O_3_ Thin Films for High-Sensitivity and Low-Dose Direct X-Ray Detection

**DOI:** 10.3390/nano15171360

**Published:** 2025-09-03

**Authors:** Lan Yang, Dingyuan Niu, Yong Zhang, Xueping Zhao, Xinxin Li, Jun Zhu, Hai Zhang

**Affiliations:** 1 School of Science, Inner Mongolia University of Technology, Hohhot 010051, China; 2 College of Materials Science and Engineering, Inner Mongolia University of Technology, Hohhot 010051, China; 3 School of Physical Science and Technology, Inner Mongolia University, Hohhot 010021, China

**Keywords:** β-Ga_2_O_3_ thin film, LPCVD, PECVD, direct X-ray detector, oxygen vacancy, high-gain, low dose detection limit

## Abstract

Ultra-wide bandgap β-Ga_2_O_3_ is a promising low-cost alternative to conventional direct X-ray detector materials that are limited by fabrication complexity, instability, or slow temporal response. Here, we comparatively investigate β-Ga_2_O_3_ thin films grown on c-sapphire by low-pressure chemical vapor deposition (LPCVD) and plasma-enhanced CVD (PECVD), establishing a quantitative linkage between growth kinetics, microstructure, defect landscape, and X-ray detection figures of merit. The LPCVD-grown film (thickness ≈ 0.289 μm) exhibits layered coalesced grains, a narrower rocking curve (FWHM = 1.840°), and deep-level oxygen-vacancy-assisted high photoconductive gain, yielding a high sensitivity of 1.02 × 10^5^ μC Gy_air_^−1^ cm^−2^ at 20 V and a thickness-normalized sensitivity of 3.539 × 10^5^ μCGy_air_^−1^ cm^−2^ μm^−1^. In contrast, the PECVD-grown film (≈1.57 μm) shows dense columnar growth, higher O/Ga stoichiometric proximity, and shallow-trap dominance, enabling a lower dark current, superior dose detection limit (30.13 vs. 57.07 nGy_air_ s^−1^), faster recovery, and monotonic SNR improvement with bias. XPS and dual exponential transient analysis corroborate a deep-trap persistent photoconductivity mechanism in LPCVD versus moderated shallow trapping in PECVD. The resulting high-gain vs. low-noise complementary paradigm clarifies defect–gain trade spaces and provides a route to engineer β-Ga_2_O_3_ thin-film X-ray detectors that simultaneously target high sensitivity, low dose limit, and temporal stability through trap and electric field management.

## 1. Introduction

X-ray detection has demonstrated a wide range of potential applications in the domains of public safety, industrial non-destructive testing, medical imaging, and material microstructure investigation [1,2,3,4]. High-efficiency X-ray detectors must simultaneously feature high density, high resistivity, low crystal defect density, etc., to achieve efficient X-ray absorption, suppress dark current, enable the detection of low-dose-rate X-rays, and improve detector response speed, and high stability is also required to extend the service life [5,6,7]. Therefore, the development of high-performance X-ray detectors is crucial for achieving accurate and fast X-ray detection.

Currently, the most extensively used X-ray detectors in the commercial market are indirect conversion detectors [8], which convert X-rays into visible light through the introduction of scintillators and then convert visible light into electrical signals using photodiodes [9,10,11]. Compared to indirect detectors, direct-type X-ray detectors do not require scintillator materials for light conversion, offering a distinct advantage in terms of higher X-ray conversion efficiency, sensitivity, spatial resolution, and lower noise levels [12,13,14,15,16,17]. However, the commonly used direct X-ray detection materials, such as silicon (Si), amorphous selenium (a-Se), cadmium zinc telluride (CdZnTe), perovskite, etc., face issues like complex manufacturing processes, high costs, and poor stability, which limit their widespread application [18,19,20,21,22,23].

In recent years, the wide bandgap semiconductor gallium oxide (Ga_2_O_3_) has emerged as a new material choice for exploring high-performance X-ray detectors due to its high density, excellent thermal and chemical stability, significant radiation resistance, and inherent rigid structure [24,25,26,27,28]. Moreover, the X-ray absorption coefficient of Ga_2_O_3_ is far higher than that of diamond, comparable to that of Si and perovskite materials, and is less susceptible to visible light interference, which gives it significant advantages in the field of X-ray detection. Early work on unintentionally doped (UID) β-Ga_2_O_3_ single crystals established device feasibility for X-ray detection but suffered slow temporal response owing to abundant oxygen vacancies (V_O_) [29]. Fe doping increased resistivity and accelerated response through compensation of donor-like defects [30]. Mg doping of a single crystal β-Ga_2_O_3_ simultaneously raised resistivity, suppressed V_O_, and delivered 16 times the sensitivity of a-Se [31]. Al alloying widened the bandgap of single-crystal β-Ga_2_O_3_, lowering intrinsic carrier density and enabling high sensitivity [32]. Thus, intentional dopants (Fe, Mg, Al) systematically couple defect suppression with resistivity/band engineering to enhance performance. Although X-ray detectors based on Ga_2_O_3_ single crystals have made significant progress, there are still certain challenges in the integration of functional modules and large-scale applications due to difficulties in growing large-sized, high-quality single crystals and the lack of effective P-type doping.

Thin-film-based X-ray detectors have emerged at the forefront of current research owing to their advantages in large-area deposition on diverse substrates, direct monolithic integration with CMOS/flexible systems, compact device size, high integration density, and reduced manufacturing costs [33]. Significant advances have been made in recent years in the growth techniques, material processing, and corresponding device performance of Ga_2_O_3_ thin films. However, current thin-film fabrication techniques in Ga_2_O_3_ film-based X-ray detection are confronted by two major challenges: the limitations inherent in film formation processes restrict the preparation of large-area, high-quality films, and the concurrent optimization of sensitivity and detection limits alongside defect engineering and crystal quality remains unrealized, severely hindering their practical implementation. In 2024, Chen et al. [34] proposed enhancing the density of Ga_2_O_3_ films through indium (In) doping, fabricating an X-ray detector based on indium-doped gallium oxide (Ga_2_O_3_:In) microwires. However, the one-dimensional structure of the microwires makes the doping process highly susceptible to limitations such as doping non-uniformity, lattice defects, interface states, and carrier transport inefficiencies, compounded by challenges in scalable fabrication. Consequently, achieving high-sensitivity, low-detection-limit X-ray detectors with this approach remains difficult. In 2024, Liang et al. [35] first employed amorphous a-Ga_2_O_3_ films in X-ray neuromorphic synapses and found that the films exhibited an oxygen vacancies/lattice oxygen ratio of 1:0.39. The resultant high concentration of oxygen vacancies enhanced interfacial electron tunneling, yielding a sevenfold increase in X-ray sensitivity with irradiation cycles (from 20.5 to 164.1 μCmGy_air_^−1^ cm^−2^). However, the amorphous structure together with the elevated oxygen vacancy density led to extremely low carrier mobility, making it difficult to balance high sensitivity with a low detection limit. Wang et al. [36] fabricated 50 nm amorphous a-Ga_2_O_3_ films via atomic layer deposition (ALD), achieving an oxygen-vacancy-related ratio O_II_/(O_I_ + O_II_) = 35.5%. The corresponding X-ray detector exhibited a sensitivity of 952 μCGy_air_^−1^ cm^−2^. Nevertheless, the inherently low ALD growth rate (0.55 Å per cycle) impedes rapid large-area fabrication; moreover, the minimal thickness inherently restricts X-ray absorption efficiency, particularly for high-energy photons. Kim et al. [37] grew 700 nm α-Ga_2_O_3_ films via hydride vapor phase epitaxy (HVPE). Metal–semiconductor–metal (MSM) detectors fabricated from these films exhibited a shift in the charge neutrality point, and their charge collection efficiency (CCE) decreased from 152.2% to 105.5% with rising X-ray dose rate. Also in 2024, Gan et al. [38] utilized MOCVD to synthesize β-Ga_2_O_3_ films and demonstrated that the growth mode transformed from island growth to step-flow by employing a 4° off-cut substrate together with a high growth temperature of 900 °C. The sensitivity reached as high as 3.72 × 10^5^ μCGy_air_^−1^ cm^−2^. This performance stems from photoconductive gain, where deep-level traps (e.g., self-trapped holes, oxygen vacancies) extend carrier lifetimes, enabling multiple electron transits before recombination. Although step-flow growth can reduce trap density, the persistence of deep-level traps still results in slow recovery dynamics, limiting applicability in fast temporal detection. Furthermore, the high cost of MOCVD and the combined requirement of MOCVD plus off-cut substrates impede compatibility with existing CMOS production lines.

Current Ga_2_O_3_ thin-film growth still lacks a route that unifies scale, defect moderation, and crystal quality, limiting device engineering application. To address these challenges, a complementary process paradigm integrating Low-Pressure Chemical Vapor Deposition (LPCVD) and Plasma-Enhanced Chemical Vapor Deposition (PECVD) was utilized to co-optimize β-Ga_2_O_3_ films in this work. We fabricated high-performance gallium oxide (Ga_2_O_3_) films and successfully constructed high-sensitivity X-ray detectors. We also investigated the underlying correlation mechanisms linking process parameters, film structure, defect properties, and device performance.

## 2. Materials and Methods

**Figure 1:**
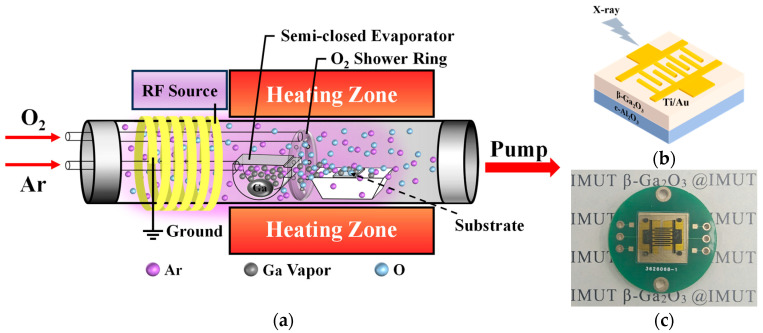
(**a**) Experimental setup for β-Ga_2_O_3_ thin film growth, (**b**) schematic diagram, and (**c**) physical image of the fabricated MSM-type detector.

Figure 1a depicts the experimental setup used for β-Ga_2_O_3_ thin film deposition [39]. Metallic gallium (Ga) was loaded into a specially designed semi-closed evaporator as the evaporation source. Argon (Ar) gas was used as the carrier gas to transport the vaporized Ga to the c-Al_2_O_3_ substrate, and oxygen (O_2_) gas was used as a reactant gas. The system was evacuated before deposition. The heating temperature was set to 900 °C to evaporate the metallic gallium (Ga) into the gas phase. Simultaneously, Ar gas and O_2_ gas were introduced into the chamber with a flow rate of 200 sccm and 6 sccm, respectively. Two different processes were used in this study: low-pressure chemical vapor deposition (LPCVD) and plasma-enhanced chemical vapor deposition (PECVD). PECVD requires additional plasma generation by radio frequency inductively coupled plasma (ICP) excitation of the reaction chamber gas. The plasma will ionize the process gas and decompose it into atomic oxygen (O*) and peroxide/superoxide free radical intermediates. These activated substances react with the gaseous gallium, and form gallium oxide (Ga_2_O_3_) on the substrate surface. In both processes, the deposition time was 60 min, and the deposition pressure was set to 80 Pa. The plasma power was set to 50 W, and other parameters remained the same as in LPCVD. The above experiments have all been verified to be repeatable.

Ti/Au (100 nm/100 nm) electrodes were deposited on the thin film surface using magnetron sputtering equipment (JGP 450G, SKY Technology Development Co., Ltd., Shengyang, China). The metal electrodes feature a finger width of 200 μm and a finger length of 3800 μm. The count of electrodes is 5 pairs (as shown in Figure 1b), and their effective illumination area is 0.0912 cm^2^. The physical photograph of the fabricated device is shown in Figure 1c. The surface and cross-section morphology of the films were observed using a scanning electron microscope(SEM) (SU 8820, Hitachi, Tokyo, Japan), and the thickness was measured from the cross-section morphology; the optical transmittance of the films was tested and the optical bandgap was calculated by a UV–vis spectrophotometer (JASCO V-760, JASCO Corporation, Tokyo, Japan); the crystalline quality and stress distribution of the films was measured by an X-ray diffractometer (XRD) (Empyrean, Malvern Panalytical, Malvern, UK); steady-state fluorescence spectrometer (Edinburgh FLS1000, Livingston, UK) was used to measure the Photoluminescence excitation spectra of the Ga_2_O_3_ thin film; and X-ray photoelectron spectroscopy (XPS) (ESCALAB 250Xi, Thermo Fisher, Waltham, MA, USA) to quantitatively analyze the elemental composition and content of the films. In X-ray detector performance testing, the X-ray source used is XH501, produced by Dandong Zhida Radiativey Co., Ltd. (Dandong, China), with a silver target, tube voltage of 40 kV, and tube current adjustment range of 0~550 μA. Since the X-ray dose rate is proportional to the tube current, it can achieve a dose rate output of 0~2312 μGy_air_/s. The X-ray dose rates corresponding to different X-ray tube currents are shown in Table 1. In all tests, X-rays were irradiated perpendicular to the β-Ga_2_O_3_ detector at an irradiation distance of 2.5 cm.

## 3. Results and Discussion

**Figure 2:**
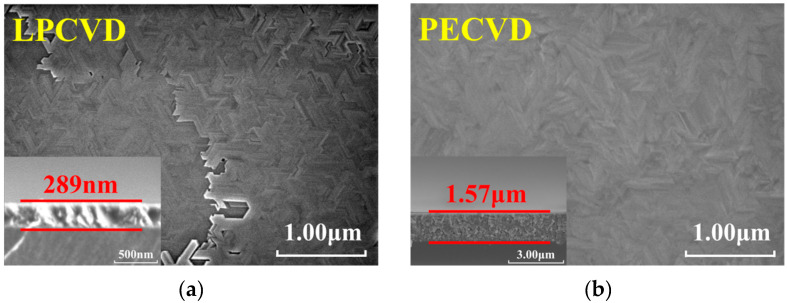
SEM surface topography; the insets show the corresponding cross-section: (**a**) LPCVD, (**b**) PECVD.

Figure 2a,b show scanning electron microscope (SEM) images of Ga_2_O_3_ thin films’ surface grown by using LPCVD and PECVD processes, respectively, and the insets show the cross-sectional morphology of the two films. It can be observed that the thin film grown by LPCVD exhibits a regular crystal structure with clear grain boundaries and surface morphology showing significant stacking phenomena, presenting a layered growth mode. The film thickness is 289 nm, with a relatively slow growth rate. Cross-sectional morphology indicates that at lower growth rates, grains merge sufficiently laterally. The thin film grown by PECVD shows no obvious pores or defects on the surface, exhibits grain boundary ridges with slight undulations, significantly improved surface density, and a quasi-columnar to columnar cross-section that continuously extended with island-like growth. The PECVD film achieved a thickness of approximately 1.57 μm within the same deposition time, with the growth rate significantly enhanced, reflecting a plasma-activated vertical growth kinetics mechanism. This indicates that plasma can introduce more active particles on the surface and promote more surface reactions, which helps optimize lattice matching and interface bonding between epitaxial films and substrates, making it easier for atoms to adsorb and nucleate on the substrate and form uniform, dense films. It also reflects the characteristics of incomplete lateral rearrangement after rapid multi-point nucleation. This difference in growth kinetics between “dense-coalescence dominated” and “rapid-columnar extension” demonstrates that different preparation processes can significantly alter the properties of β-Ga_2_O_3_ films.

**Figure 3:**
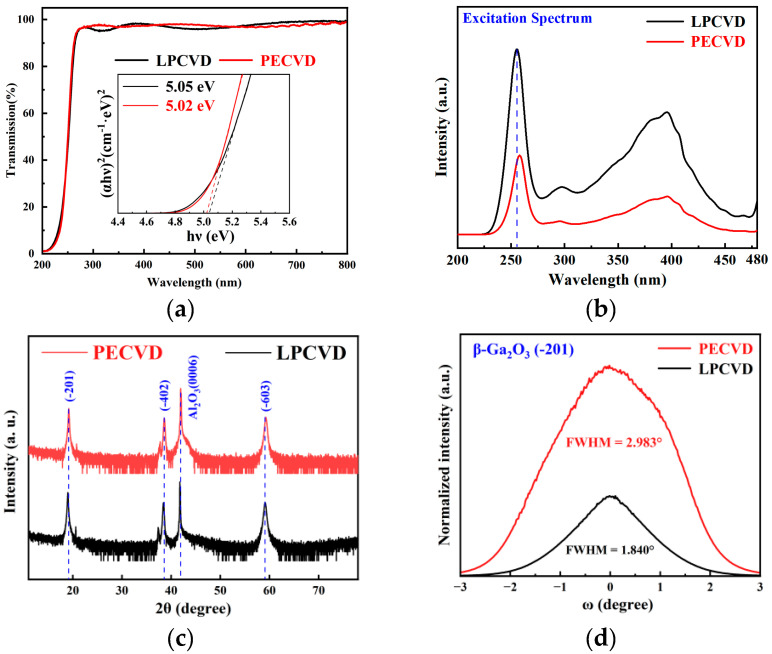
(**a**) Transmittance spectra and calculated optical bandgap of β-Ga_2_O_3_ thin films grown by LPCVD and PECVD. (**b**) Photoluminescence excitation spectra of the films grown via LPCVD and PECVD. (**c**) X-ray diffraction spectra. (**d**) Rocking curve of (−201) crystal plane of β-Ga_2_O_3_ thin films.

Figure 3a depicts the transmittance of the gallium oxide films grown by using the two processes. The incident light transmittance of both samples exceeds 90% in the visible wavelength range. To further analyze the optical bandgap properties of the thin films more extensively, the Tauc equation was used as follows [40]:

(1) $${\left(\frac{\alpha h\nu }{A}\right)}^{2}\;=\;h\nu \;-\;E_{g},$$

(2) $$\alpha \;=\;\left(1/d\right)\ln\left(1/T\right),$$

where *α* is the absorption coefficient; *h* is Planck’s constant; *ν* is the frequency of the incident photon; *d* is the thickness of the film; and *T* is transmittance. Combined with the transmission spectra of the film, the horizontal coordinate is *hν* and the vertical coordinate is (*αhν*)^2^; a straight line can be obtained by linearly fitting the absorption mutation data. Through extrapolation of the linear region, the optical bandgap *E_g_* of the film can be calculated, as shown in the inset. The band gap of the LPCVD-grown film is 5.05 eV; the band gap of the PECVD-grown film is 5.02 eV. *E_g_* of PECVD-grown film is slightly higher, possibly due to fitting error or indicating weaker Urbach tail states/shallow defect absorption and fewer surface/bulk defect recombination centers. 

Figure 3b presents the photoluminescence excitation (PLE) spectra of the films grown via LPCVD and PECVD. Distinct excitation peaks appear at 255 nm and 258 nm for the LPCVD and PECVD samples. Using Formula (3), the bandgap energies were calculated to be 4.87 eV and 4.81 eV [41], respectively. These values, corroborated by the bandgap values extracted using the Tauc equation, confirm that the LPCVD-grown film possesses a marginally larger bandgap than its PECVD counterpart.

(3) $$E_{g}\;=\;\frac{hc}{\lambda }$$

Here, *h* represents the Planck constant; *c* is the speed of light in a vacuum; and *λ* is the peak wavelength of the excitation spectrum.

Figure 3c shows the XRD characterization of the films. The Bragg diffraction peaks corresponding to (−201), (−402), and (−603) crystals originating from β-Ga_2_O_3_ can be observed, indicating that the films are all growing preferentially in the direction of (−201). However, there are no obvious diffraction signals except for the diffraction peaks of the above crystal planes, which shows that the grown gallium oxide thin films are pure phase β-Ga_2_O_3_. In order to further analyze the crystallinity of β-Ga_2_O_3_ thin films, the (−201) crystal plane diffraction peak in XRD was analyzed. It was found that the full width at half maximum (FWHM) of the Ga_2_O_3_ thin film grown by LPCVD was 0.147°, and the FWHM of the Ga_2_O_3_ thin film grown by PECVD was 0.173°. This indicates that the thin films have good crystallinity, and the β-Ga_2_O_3_ thin film grown by PECVD has smaller grain size and a more refined structure.

In order to characterize the orientation dispersion and crystal quality of the β-Ga_2_O_3_ thin film, high-resolution XRD rocking curve measurements were performed on the (−201) crystal plane of the films, as shown in Figure 3d. The FWHM of the rocking curve for the film grown by LPCVD was measured to be 1.840°, while the FWHM of the rocking curve for the films grown by PECVD was 2.983°. Thus, it can be seen that β-Ga_2_O_3_ thin films exhibit high crystallinity and orientation. Meanwhile, the FWHM of the rocking curve of β-Ga_2_O_3_ thin film grown by PECVD is wider than that of LPCVD, indicating larger grain tilt and mosaic spread in PECVD-grown films. Combined with SEM data, the larger FWHM is attributed to the high initial nucleation density induced by highly active oxygen in PECVD, insufficient lateral coalescence, and rapid vertical advancement along with columnar interface stress freezing, which jointly restrict tilt reduction.

We employed the Scherrer equation to calculate the crystallite sizes of the thin films grown via LPCVD and PECVD based on the full width at half maximum (FWHM) of the rocking curves, yielding values of 4.51 nm and 2.77 nm, respectively. The crystallite size is inversely proportional to the FWHM of the rocking curve: a smaller crystallite size corresponds to a larger FWHM, while a larger crystallite size results in a narrower FWHM. This relationship is consistent with the FWHM values derived from the rocking curve analysis.

**Figure 4:**
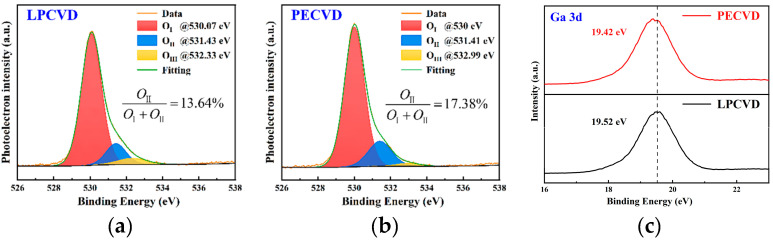
XPS spectra of β-Ga_2_O_3_ thin films. (**a**) O 1s core-level spectra of β-Ga_2_O_3_ thin film grown by LPCVD. (**b**) O 1s core-level spectra of β-Ga_2_O_3_ thin film grown by PECVD. (**c**) Comparison of Ga 3d core-level spectra.

The chemical states of elements in films grown by two processes were investigated by X-ray photoelectron spectroscopy (XPS). All spectra were calibrated against the reference C 1s peak at 284.8 eV, and the binding energy uncertainty is approximately ±0.05 eV. For quantitative analysis of oxygen vacancy (V_O_) defect concentration, XPS tests were conducted on the O 1s energy level followed by Gaussian fitting peak analysis, as shown in Figure 4a,b. The O 1s spectra can be divided into three characteristic peaks: O_I_ corresponds to lattice oxygen of the O-Ga bond in Ga_2_O_3_, O_II_ represents oxygen vacancy defects in the oxide, and O_Ⅲ_ mainly originates from surface chemical adsorbed substances (such as adsorbed O_2_, OH^−^, etc.). In the film grown by LPCVD, three peaks are located at 530.07 eV, 531.43 eV, and 532.33 eV, respectively; in the film grown by PECVD, three peaks are located at 530 eV, 531.41 eV, and 532.99 eV. Since O_II_/(O_I_ + O_II_) reflects the concentration of oxygen vacancies, the calculation results show that the oxygen vacancy concentrations in the films grown by LPCVD and PECVD are 13.64% and 17.38%, respectively. Although the O_I_/(O_I_ + O_II_) ratio of PECVD samples is higher than that of LPCVD, it is important to note that PECVD can introduce plasma-activated oxygen (O*, peroxy/superoxide intermediates) and surface hydroxy groups, which are prone to produce overlapping in the O_II_ energy level region. This component includes transient activated adsorption and reoxidation transition state and is not entirely equivalent to an increase in stable lattice oxygen vacancies as deep defects. Furthermore, during subsequent mild annealing, they can be further incorporated into the lattice, thereby reducing the density of steady-state oxygen vacancies.

According to the XPS spectra of Ga 3d, shown in Figure 4c, the binding energy corresponding to Ga 3d in the LPCVD-grown film is 19.52 eV, and the binding energy of Ga 3d in the film grown by PECVD is 19.42 eV. The Ga 3d main peak is located in the 19.4–19.5 eV range under both processes, corresponding to the Ga^3+^-O bond. Compared with LPCVD, the peak position of PECVD samples slightly shifts towards lower binding energy by approximately 0.10 eV (this magnitude exceeds the uncertainty limit of ±0.05 eV for energy calibration and differential charging). This can be attributed to the subtle redistribution of local chemical/stress environments and differences in electron screening brought about by rapid plasma oxidation or possibly due to more complete oxidation, reducing suboxide clusters leading to local potential field homogenization, suggesting that PECVD films possess more uniform local potentials and lower defect concentrations.

To gain a deeper understanding of the impact of oxygen vacancies on the overall chemical state of materials, Formula (4) was used to calculate the atomic ratio of O to Ga. The peak areas of Ga 3d and O 1s (*I*_*O* 1*s*_, *I*_*Ga* 3*d*_) were extracted from the XPS data, then normalized by dividing by the elemental sensitivity factors (*S*_*O* 1*s*_ = 0.711, *S*_*Ga* 3*d*_ = 0.34), and, finally, the ratio of the normalized areas is the atomic ratio of O to Ga [42,43].

(4) $$O/Ga\;=\;\frac{I_{O1s}/S_{O1s}}{I_{Ga\;3d}/S_{Ga\;3d}},$$

According to calculations, the O/Ga atomic ratio (PECVD: 1.343; LPCVD: 1.298) was obtained. The PECVD sample is closer to the ideal stoichiometry of Ga_2_O_3_ (O/Ga = 1.5), indicating that the PECVD sample is more fully oxidized and has more complete chemical coordination. The reduction in a small amount of oxygen vacancies not only reduces the mid-gap defect states, which is beneficial for suppressing carrier scattering and non-radiative recombination, but also reduces the local stress and structural disorder induced by point defects, thereby improving the crystal quality.

**Figure 5:**
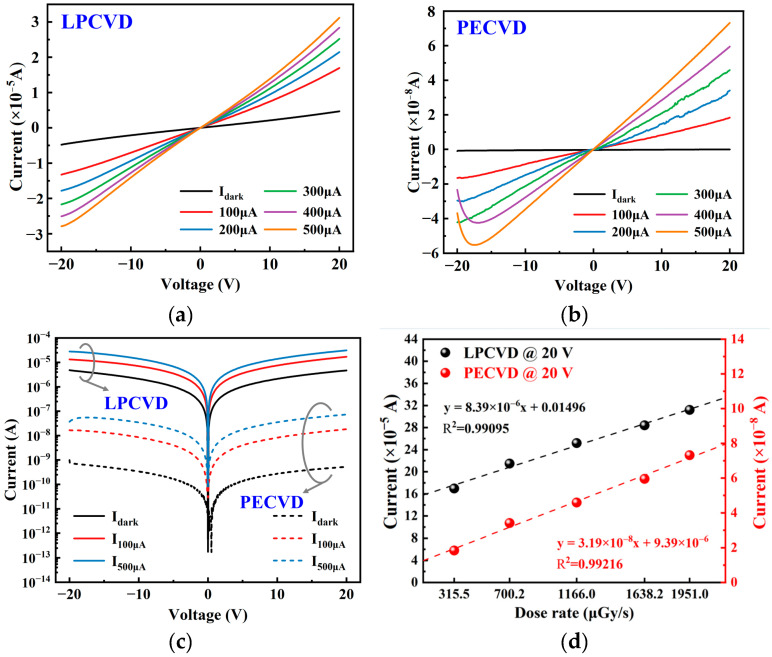
I–V characteristic curves of the detector under X-ray irradiation at different tube currents: (**a**) LPCVD; (**b**) PECVD; (**c**) logarithmic I–V characteristics of the detector in dark state and under X-ray irradiation at room temperature; (**d**) dose-rate dependent photocurrent of β-Ga_2_O_3_ MSM X-ray detectors fabricated by LPCVD and PECVD at 20 V bias.

Two samples were fabricated into MSM structure X-ray detectors (Figure 1b,c). As shown in Figure 5a,b, both β-Ga_2_O_3_ MSM devices (LPCVD and PECVD) exhibited near-linear I–V characteristics under X-ray irradiation with tube voltage of 40 kV and tube current of 100–500 μA, as well as in dark state, indicating good ohmic contact at the metal/semiconductor interface. The current of both types of devices increases almost linearly with bias voltage under X-ray irradiation, indicating low interface barriers and minimal carrier injection limitations. Based on inter-band recombination theory, the recombination probability of carriers generated under X-ray irradiation increases with the carrier density and is proportional to the incident X-ray dose rate [44]. When the tube current is 400 μA and 500 μA, the photocurrent of the X-ray detector fabricated by PECVD exhibits a slight inflection characterized by “initial slight decline followed by rebound” as the bias voltage increases. This phenomenon can be attributed to the temporary limitation of effective mobility or lifetime caused by competitive filling of traps (partially deep/intermediate energy levels) in the moderate bias region. At higher electric fields, the drift time shortens rapidly, traps tend to saturate, and photoconductive gain is restored and enhanced.

The logarithmic I–V characteristics of the β-Ga_2_O_3_-based X-ray detector in dark state and under X-ray irradiation at room temperature are shown in Figure 5c. Under X-ray irradiation, the detector current significantly increases, which is related to the generation of photoelectron-hole pairs and secondary electrons [45]. In comparison, the X-ray detector fabricated by PECVD exhibits lower dark current, indicating that shallow energy level traps dominate and reduce leakage caused by thermal excitation or slow recombination; its photocurrent increases more smoothly with dose rate (tube current), demonstrating electrical stability and uniformity of defect distribution.

Figure 5d shows the relationship between dose rate and X-ray response current for β-Ga_2_O_3_ MSM detectors fabricated by LPCVD and PECVD under a bias voltage of 20 V. The photocurrent of the device fabricated by LPCVD is on the order of 10^−5^ A, approximately 10^3^ times higher than that of PECVD (10^−8^ A), and both exhibit an approximately linear relationship within this dose rate range, *I*_*X-ray*_∝*kD*^1^, indicating a balance between carrier recombination and generation. The higher slope suggests that the detector fabricated by LPCVD has a stronger trap-assisted photoconductive gain, while the detector fabricated by PECVD demonstrates lower gain but shows potential for faster and more stable response.

**Figure 6:**
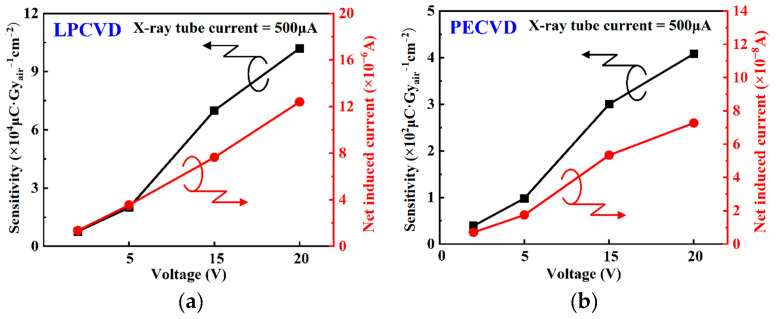
The variation in net induced current and sensitivity at different bias voltages of 2 V, 5 V, 15 V, and 20 V at a fixed X-ray tube current of 500μA: (**a**) LPCVD, (**b**) PECVD.

Figure 6a,b present the variations in net induced current and sensitivity under different biases (2, 5, 15, 20 V) at a fixed X-ray tube current of 500 μA. Sensitivity is defined as follows [8]:

(5) $$S\;=\;\frac{I_{X\text{-}ray}\;-\;I_{dark}}{D\cdot A},$$

where *I_X-ray_* is the photocurrent generated by X-ray irradiation; *I_dark_* is the dark current; *D* is the X-ray dosage rate; and *A* is the effective detection area. As demonstrated in Figure 6a,b, the net induced current (*I_X-ray_* − *I_dark_*) and sensitivity rise monotonically as the bias voltage increases, reflecting the synergistic improvement of carrier collection efficiency and photoconductive gain by electric field enhancement (derived from the high electric field reducing *τ_T_* and improving the carrier collection efficiency) [31]. At a bias voltage of 20 V, the LPCVD-fabricated detector achieved a sensitivity of 1.02 × 10^5^ μCGy_air_^−1^ cm^−2^, significantly higher than the 408 μCGy_air_^−1^ cm^−2^ of the detector fabricated by PECVD. This difference may be related to potential deep traps extending carrier lifetime and thus amplifying photoconductive gain, may also include composite effects from differences in thickness and absorption path.

To eliminate geometric and thickness (*d*) factors, normalized sensitivity (*S_norm_*) is defined as follows:

(6) $$S_{norm}\;=\;S/d$$

After separating the thickness factor, it is similarly observed that the normalized sensitivity of the LPCVD-fabricated detector reached 3.539 × 10^5^ μCGy_air_^−1^ cm^−2^ μm^−1^ at a bias voltage of 20 V, significantly higher than the 272 μCGy_air_^−1^ cm^−2^ μm^−1^ of the PECVD-fabricated detector, indicating that the intrinsic material quality of LPCVD-grown films is superior to PECVD-grown films. Although the sensitivity of devices fabricated by PECVD is relatively low, their curves exhibit no obvious saturation inflection point, indicating that the defects induced by the plasma process are shallower and more orderly distributed, which can realize the linear regulation of sensitivity by bias voltage.

**Figure 7:**
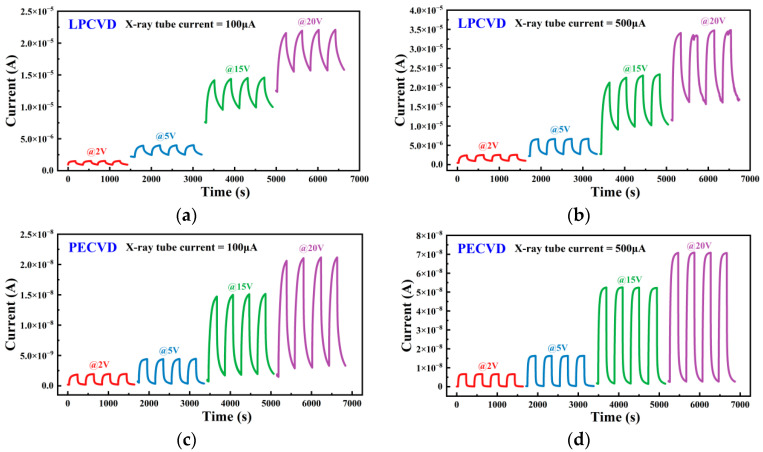
Transient response characteristics (I–t) of the X-ray detectors fabricated by (**a**,**b**) LPCVD and (**c**,**d**) PECVD at bias voltages of 2, 5, 15, and 20 V. X-ray tube currents are 100 μA for (**a**,**c**) and 500 μA for (**b**,**d**). The different colored lines in each figure represent the response characteristics at 2 V, 5 V, 15 V, and 20 V bias, respectively.

Figure 7, respectively, shows the I–t response characteristics (transient response characteristics) of X-ray detectors fabricated by LPCVD and PECVD at different X-ray tube currents (100 and 500 μA) and bias voltages. As shown in Figure 7, PECVD device maintain an extremely low baseline and smaller drift, quickly entering a steady state and maintaining a flat platform, demonstrating better temporal stability and baseline reproducibility. LPCVD device response curves show slight time-dependent rise. These differences may originate from defect states and interface barrier variations caused by deposition processes: higher carrier density and lower contact barriers form in LPCVD devices, enhancing drift current while amplifying dark current; leakage current is effectively suppressed due to higher barriers or lower shallow donor concentrations in PECVD devices. The photocurrent of both detectors increases with the increase in X-ray tube current. Meanwhile, under strong electric field, the photocurrent increases significantly with the increase in bias voltage. This is related to the high carrier collection efficiency of devices under strong electric fields, where photoconductivity gain can be defined as *G* = *τ_l_*/*τ_T_*, where *τ_T_* = *L*/*μE* (*L* is the electrode spacing, *μ* is the carrier mobility, *E* is the electric field), and τₗ is the carrier lifetime [46]. Intrinsic defects in Ga_2_O_3_ (such as oxygen vacancies) can extend the carrier lifetime *τ_l_*, thereby increasing G. At the same time, the increase in electric field strength reduces *τ_T_*, leading to an increase in G, which significantly enhances the photocurrent as the bias increases.

**Figure 8:**
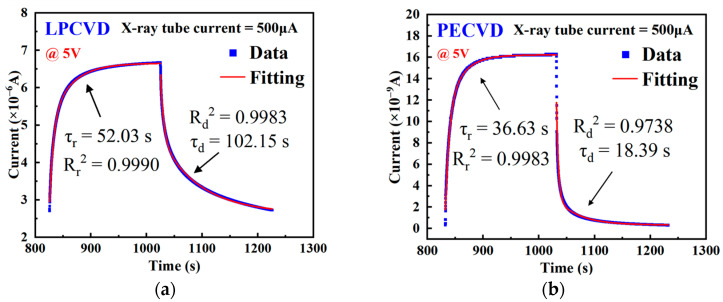
Time-resolved photocurrent of MSM β-Ga_2_O_3_ X-ray detectors fabricated by (**a**) LPCVD and (**b**) PECVD at a tube current of 500 μA and bias of 5 V.

The photocurrent response time dependency curves of β-Ga_2_O_3_ X-ray detectors fabricated by LPCVD and PECVD under an X-ray tube current of 500 μA and a 5 V bias are shown in Figure 8a,b, respectively. The response time (*τ_r_*) and recovery time (*τ_d_*) were calculated by double exponential fitting of the I–t characteristic curves [34]:

(7) $$I\left(t\right)\;=\;I_{0}\;+\;A_{1}e^{-t/\tau_{r}}\;+\;A_{2}e^{-t/\tau_{d}},$$

Both types of devices exhibit varying degrees of response/recovery hysteresis, indicating the presence of persistent photoconductivity (PPC). Both types of devices exhibit different degrees of response/recovery hysteresis, indicating the presence of persistent photoconductivity (PPC). Under X-ray excitation, high-energy electrons not only directly generate electron-hole pairs [42] but also promote the multi-step ionization of V_O_^0^ state oxygen vacancies to V_O_^1+^ and V_O_^2+^ states, possibly forming more positively charged deep capture centers Vo^3+^. These deep positive centers inhibit hole refilling and delay recombination, resulting in long-lifetime tails and slow recovery [24,47,48,49,50]. Since the formation and stepwise ionization of V_O_^0^ state oxygen vacancies require a longer time to reach dynamic equilibrium, the improvement in response speed is limited. Devices fabricated by LPCVD exhibit higher steady-state photocurrent and slower response/recovery times (*τ_r_* = 52.03 s, *τ_d_* = 102.15 s), reflecting persistent photoconductivity caused by deep traps, whereas devices fabricated by PECVD have lower steady-state photocurrent but faster response/recovery time (*τ_r_* = 36.63 s, *τ_d_* = 18.39 s), which also indicates that the defects in PECVD films are dominated by shallow traps. The R^2^ values for the double-exponential fitting of all samples are greater than 0.97, indicating a very high degree of agreement between the fitting curves and the experimental data. This can indirectly confirm the credibility of the carrier lifetime parameters.

**Figure 9:**
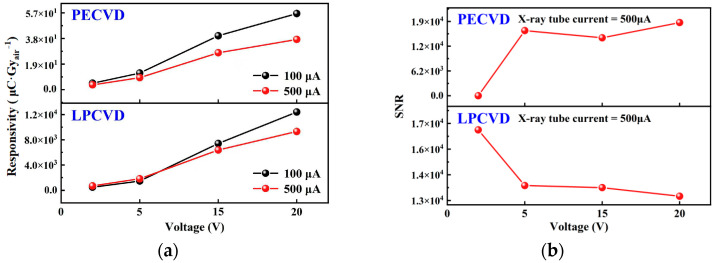
(**a**) The responsivity of the detectors fabricated by LPCVD and PECVD varies with the bias voltage under X-ray irradiation at different tube currents. (**b**) The signal-to-noise ratio (SNR) of the detectors fabricated by LPCVD and PECVD varies with the bias voltage at an X-ray tube current of 500 μA.

In order to study the dose-dependent photoresponse characteristics of Ga_2_O_3_-based X-ray detectors, the responsivity of the device under different X-ray dose rates was calculated, and the influence of bias voltage on the responsivity was investigated, as shown in Figure 9a. Responsivity is defined as the ratio of net photocurrent to incident dose rate [44]:

(8) $$R\;=\;\frac{I_{X\text{-}ray}\;-\;I_{dark}}{D},$$

As shown in Figure 9a, the responsivity *R* of both detectors increases with increasing bias. However, as the tube current increases (i.e., the dose rate increases), the responsivity of both X-ray detectors slightly decreases. This is because the increased number of carriers excited by high-dose X-rays leads to a higher probability of carrier recombination, reflecting the sublinear gain compression due to elevated recombination probability at high carrier density.

The SNR can be expressed using the formula [25]:

(9) $$SNR\;=\;\frac{I_{signal}}{I_{noise}},$$

(10) $$I_{signal}\;=\;{\bar{I}}_{X\text{-}ray}\;-\;{\bar{I}}_{dark},$$

(11) $$I_{\mathrm{noise}}\;=\;\sqrt{\frac{1}{N}\sum_{i}^{N}{\left(I_{i}\;-\;{\bar{I}}_{X\text{-}ray}\right)}^{2}},$$

where ${\bar{I}}_{X\text{-}ray}$ is the average photocurrent under X-ray irradiation; ${\bar{I}}_{dark}$ is the average dark current; *N* is the number of photocurrent data points under X-ray irradiation; *i* is an integer ranging from 1 to *N*; and *I_i_* is the irradiation steady-state photocurrent sampling point.

The SNR of the device fabricated by PECVD and LPCVD shows opposite trends with increasing bias in Figure 9b. This suggests that the emission/recapture fluctuation of deep traps (G-R and 1/f noise components) in LPCVD devices at high fields increases faster than the optical signal gain and also indicates that there may be more deep-level traps in the thin film grown by LPCVD. It also indicates that there may be more deep-level traps present in the thin films grown by LPCVD. In contrast, devices fabricated by PECVD are dominated by shallow traps, and the noise increases more slowly with the bias voltage.

According to the International Union of Pure and Applied Chemistry (IUPAC), the low dose rate detection limit (*LoD*_min_) corresponding to an SNR of three generated by X-ray detectors is considered its detection limit.

(12) $$LoD_{\min}\;=\;\frac{3I_{noise}}{S\cdot A}$$

The detection limit *LoD*_min_ of the detector fabricated by LPCVD is calculated from the above equation to be 57.07 nGy_air_ s^−1^, which is significantly higher than 30.13 nGy_air_ s^−1^ of the detector fabricated by PECVD. These suggest that the detector fabricated by PECVD has greater advantages in scenarios requiring precise detection of low doses or a wide dynamic range under high bias voltage. Moreover, it has a shorter recovery time, lower noise, and is better suited for applications in rapid measurement and stable calibration scenarios.

The comprehensive performance of various X-ray detectors with different preparation processes and equivalent thicknesses in recent years is summarized in Table 2. Compared to β-Ga_2_O_3_ or other semiconductor X-ray detectors in the literature that rely on hundreds to thousands of micrometers of thickness and high reverse bias to achieve limited sensitivity, this work achieves a high normalized sensitivity of 3.539 × 10^5^ μCGy_air_^−1^ cm^−2^ μm^−1^ under only 20 V bias with ultra-thin film (≈0.29 µm), while PECVD film maintains a low detection limit of 30.13 nGy_air_ s^−1^ at low bias voltage.

## 4. Conclusions

In summary, β-Ga_2_O_3_ thin films were grown on c-plane sapphire by using LPCVD and PECVD techniques, and MSM-type X-ray detectors were fabricated. It is demonstrated that the films grown by LPCVD were thin (289 nm), where slower surface kinetics favor lateral grain coalescence and reduced mosaic tilt (narrower rocking curve), while PECVD plasma activation induces rapid multi-site nucleation and columnar advancement, increasing tilt dispersion yet improving thickness scalability and film densification.

Both films are single-phase, (−201) oriented β-Ga_2_O_3_. The film grown by PECVD yields a higher O/Ga atomic ratio (closer to stoichiometric) and a slight Ga 3d binding energy shift, indicating more uniform local electrostatic environments and a shallower trap ensemble relative to the deeper trap population inferred for LPCVD.

For the LPCVD-grown film, despite its submicron thickness, it achieves high (normalized) sensitivity (3.539 × 10^5^ μCGy_air_^−1^ cm^−2^ μm^−1^ at 20 V) through deep trap-extended carrier lifetime, which boosts photoconductive gain (*G* = *τ_l_*/*τ_T_*). However, it exhibits larger persistent photoconductivity, slower decay (*τ_d_* = 102.15 s), and SNR degradation at high bias due to trap emission/retrapping noise. The device fabricated by PECVD exhibits superior calibratability and application predictability owing to its lower dark current, linear bias scalability of SNR, faster transient (*τ_d_* = 18.39 s), and superior minimum detectable dose (30.13 nGy_air_ s^−1^), making it preferable for low-dose scenarios. Dual exponential transient fits and XPS oxygen sub-peak ratios support a model where progressive oxygen vacancy ionization V_O_^0^→V_O_^1+^/V_O_^2+^ under X-ray fields sustain long-lived photoconductivity in deep-trap dominant films; conversely, limiting deep trap density shifts operation toward rapid, low-noise regimes.

These insights motivate a hybrid optimization path: moderating deep trap density via post-oxidation and mild compensatory dopants while preserving sufficient shallow traps for controlled gain may reconcile sensitivity and speed. Meanwhile, it is also demonstrated that precise control of deposition processes and trap engineering can achieve competitive dose rate response within limited thickness, laying the foundation for subsequent pixilation and low-power array integration in medical imaging, industrial inspection, and other field applications.

## Figures and Tables

**Table 1:** X-ray dose rate corresponding to different X-ray tube currents.

Item	Value				
X-ray tube voltage (kV)	40				
X-ray tube current (μA)	100	200	300	400	500
X-ray dose rate (μGy/s)	315.5	700.2	1166.0	1638.2	1951.0

**Table 2:** Comparison of comprehensive performance of various X-ray detectors with different methods and equivalent thicknesses.

Materials	Growth Method	Device Type	Thickness (μm)	*S_norm_* (μCGy_air_^−1^ cm^−2^ μm^−1^)	*LoD_min_* (nGy_air_ s^−1^)	Ref.
β-Ga_2_O_3_	LPCVD	MSM	0.289	3.539 × 10^5^ @ 20 V	57.07	this work
β-Ga_2_O_3_	PECVD	MSM	1.5	272 @ 20 V	30.13	this work
MAPbI_3_/MAPbBr_3_	Solution Method	MSM	1060	8.197 @ 60 keV	/	[10]
Sb_1.9_Bi_0.1_S_3_	Sol–gel Method	p-i-n	4000	0.625 @ −20 V	89	[51]
α-Ga_2_O_3_	ALD	MSM	0.05	1.904 × 10^4^ @ 15 V	11.23	[36]
α-Ga_2_O_3_	HVPE	MSM	0.7	19.57 @10 V	/	[37]
α-Ga_2_O_3_	RF magnetron sputtering	MSM	0.36	4.56 × 10^5^ @ 10 V	/	[35]
β-Ga_2_O_3_	MOCVD	MSM	0.258	1.442 × 10^6^ @ 40 V	/	[38]
β-Ga_2_O_3_	MOCVD	MSM	0.319	3.86 × 10^5^ @ 50 V	/	[52]
β-Ga_2_O_3_ Microwire	CVD	MSM		5.9 × 10^5^ @ 20 V	67.4	[34]
UID β-Ga_2_O_3_	Guided-mode Method	Vertical Schottky diode	400	0.165 @ −15 V	/	[31]
β-Ga_2_O_3_:Mg	Float-Zone Growth	MSM	1000	0.339 @ −1000 V	<6.95 × 10^4^	[31]
β-Ga_2_O_3_:Al	Optical Floating Zone	MSM	1000	0.852 @ 350 V	9.8 × 10^3^	[32]

## Data Availability

The datasets generated and/or analyzed during the current study are available from the corresponding authors upon reasonable request.
